# Variation in the Fatty Acid Synthase Gene (*FASN*) and Its Association with Milk Traits in Gannan Yaks

**DOI:** 10.3390/ani9090613

**Published:** 2019-08-27

**Authors:** Bingang Shi, Yanyan Jiang, Yanli Chen, Zhidong Zhao, Huitong Zhou, Yuzhu Luo, Jiang Hu, Jon G.H. Hickford

**Affiliations:** 1 Faculty of Animal Science and Technology & Gansu Key Laboratory of Herbivorous Animal Biotechnology, Gansu Agricultural University, Lanzhou 730070, China; 2 Gene-Marker Laboratory, Faculty of Agriculture and Life Sciences, Lincoln University, Lincoln 7647, New Zealand

**Keywords:** FASN, milk traits, *Bos grunniens*, variation, yak

## Abstract

**Simple Summary:**

The yak (*Bos grunniens*) is a symbolic animal living in alpine climates (between 2000 to 5000 m) in the Qinghai–Tibetan Plateau. More than 13 million domestic yaks provide the basic resources (such as meat, milk, hair, transportation, and dung for fuel) necessary for Tibetans and nomads in China. While yak milk production is not elevated, yak milk is superior to dairy cow milk in nutrient composition (protein and fat). Fatty acid synthase (FASN) is an enzyme involved in the synthesis of fatty acids (FA) and plays a central role in de novo lipogenesis in mammals. However, there have been few reports on the effects of the FASN gene on the milk traits of yak. Our study elucidated the tissue expression of the yak *FASN* gene and the association of variants and haplotypes in the gene with milk fat percentage and total milk solid percentage. The results provide guidance for the molecular-assisted selection of milk traits in yaks.

**Abstract:**

Fatty acid synthase (FASN) is an enzyme involved in the synthesis of fatty acids (FA) and plays a central role in de novo lipogenesis in mammals. This study was conducted to ascertain the relative level of expression of the FASN gene (*FASN*) in tissues from the yak (*Bos grunniens*), and to search for variation in two regions of yak *FASN* using polymerase chain reaction single-stranded conformational polymorphism (PCR-SSCP) analyses; it also ascertains whether that variation is associated with yak milk traits. The gene was found to be expressed in twelve tissues, with the highest expression detected in the mammary gland, followed by subcutaneous fat tissue. Two regions of the gene were analyzed in 290 Gannan yaks: A region spanning exon 24-intron 24 and a region spanning exon 34. These regions both produced two PCR-SSCP patterns, which, upon sequencing, represented different DNA sequences. This sequence variation resulted from the presence of three nucleotide substitutions: c.4296+38C/T (intron 24), c.5884A/G, and c.5903G/A, both located in exon 34. The exon 34 substitutions would result in the amino acid substitutions p.Thr1962Ala and p.Gly1968Glu if expressed. Four haplotypes spanning from the exon 24-intron 24 region to exon 34 were identified. Of these, two were common (*A_1_-A_2_* and *B_1_-A_2_*), and two were rare (*A_1_-B_2_* and *B_1_-B_2_*) in the yaks investigated. The presence of *A_1_-A_2_* was associated with an increase in milk fat content (*p* = 0.050) and total milk solid content (*p* = 0.037), while diplotype *A_1_-A_2_*/*B_1_-A_2_* had a higher milk fat content (*p* = 0.038) than the other diplotypes. This study suggests that further characterization of the FASN gene might provide for an improved understanding of milk traits in yaks.

## 1. Introduction

Yaks (*Bos grunniens*) are widely distributed in alpine and sub-alpine regions (altitudes between 2000 and 5000 m) of the Qinghai–Tibetan Plateau Region of China. Their population is estimated to exceed 13 million in China alone [1] and they are a source of meat, milk, and hair. People in the Qinghai–Tibetan region consume yak milk and its products which is an important source of income for farmers. The Gannan yak (one of the twelve officially recognized domestic Chinese yak breeds) is dispersed in highland areas (greater than 2800 m above sea level) in the Gannan Region of Gansu Province, Northwest China. These yaks graze on natural pastures, but with some supplementary feeding in the dry season. As of 2018, the Gannan Tibetan Autonomous Prefecture had approximately 820,000 yaks [1]. The milk has a high fat content (5.5%–7.5% *w*/*v*) and high protein content (4.0%–5.9% *w*/*v*) at the peak of lactation [2,3], as compared to *Bos taurus* (European dairy cow) milk. Compared to *B. taurus* milk, yak milk is also richer in essential amino acids and minerals (except phosphorus) [4], and it has a higher cholesterol and sphingomyelin content [5].

Fatty acid synthase (FASN) is an enzyme component of the lipoprotein in cells, and it plays a central role in de novo lipogenesis in mammals [6]. In the human protein atlas (https://www.proteinatlas.org/ENSG00000169710-FASN/tissue#gene_information), the gene is predominantly expressed in human adipose tissue and the enzyme is predominantly found in breast and adipose tissue. The FASN enzyme has seven active sites that help catalyze the reactions that convert acetyl-CoA and malonyl-CoA to palmitate [7]. Palmitic acid is a long-chain fatty acid commonly found in bovine milk fat and adipose tissue, especially subcutaneous fat. The β-ketoacyl reductase (KR) and thioesterase (TE) domains of FASN are very close to each other in the mature protein [8]. Amino acid substitutions in the KR domain are reported to affect the function of the TE domain by altering the binding of the substrate to the region, and this can affect the fatty acid composition of muscle [9].

In humans, visceral adipose tissue expression of the FASN gene (*FASN*) is correlated with the visceral fat area, fasting plasma insulin levels, serum concentrations of IL-6, leptin and retinol-binding protein 4 (RBP4), and inversely with measures of insulin sensitivity [10]. In *B. taurus* cattle, variation in *FASN* has been found to be associated with a variety of milk traits, including milk-fat content, total milk solid content, peak milk production, milk C14 fatty acid (FA) index, and overall fatty acid (FA) composition [11,12,13,14,15]; in sheep, the expression of *FASN* is related to an increase in conjugated linoleic acid (CLA) in dairy products [16]. Finally, in pigs, variation in *FASN* has been reported to be associated with both fat and meat traits [17]. Together, these findings suggest that *FASN* plays an important role in FA metabolism.

While the yak FASN gene is not fully characterized, a GenBank sequence is available (NW-005395032). The *B. taurus* gene is located on chromosome 19 and is 18,824 base pairs in length, with 42 exons, and 41 introns [8]. The yak genome sequence draft was released in 2012 [18], and Chu et al. [19] revealed associations between single-nucleotide polymorphisms in *FASN* and meat quality traits in Datong yaks. In this context, the aim of this study was first to ascertain where yak *FASN* was expressed. Next, we searched for variation in *FASN*, and having found variation, we then ascertained whether it was associated with variation in yak milk traits.

## 2. Materials and Methods

### 2.1. Yaks Investigated and Data Collection

The animal experiments were carried out in accordance with the guidelines from the Gansu Agricultural University Animal Care Committee (2006-398).

Blood samples (*n* = 290) were collected onto FTA cards (Whatman BioScience, Middlesex, UK) from lactating Gannan yaks. These yaks were farmed on a single, large property in the Gannan–Tibetan Autonomous Prefecture (Gansu, China), and ranged in age from four to ten years old. They were collected from a variety of family groups so as to maximize diversity, and they were all grazed outdoors on pasture under the same feeding and management conditions.

Milk samples were collected from these yaks and their group and parity were recorded. Milk traits (milk protein, milk fat, milk lactose, non-fat solid, and total solid content) were analyzed using a MilkoScan™ FT1 analyser (FOSS, Hillerød, Denmark), in the Animal Quality Supervision and Testing Center of the Ministry of Agriculture of Lanzhou, China.

Twelve tissues, including spleen, liver, *longissimus dorsi* muscle, large intestine (colon), rumen, abomasum, kidney, lung, small intestine (jejunum), heart, subcutaneous fat, and mammary gland, were collected from three, five-year-old female Gannan yaks within 30 min of slaughter in October. This month is considered to be the beginning of the cold season on the Qinghai–Tibetan Plateau and typically corresponds to mid lactation as yaks calve from April to June every year, and the average lactation period is 170 days. All the tissue samples were immediately placed in liquid nitrogen and stored at −70 °C.

### 2.2. Polymerase Chain Reaction (PCR) Amplification and Single-Stranded Conformational Polymorphism (SSCP) Analysis

DNA for analysis was purified from the FTA cards using a procedure described by Zhou et al. [20]. Two different regions of yak *FASN* were chosen for analysis, with one region spanning exon 24-intron 24 and the other spanning exon 34. These regions were chosen because they were previously identified to be of importance in bovine *FASN* association studies [11]. Based on a published yak *FASN* sequence (GenBank accession no. NW_005395032.1), two sets of PCR primers were designed and their sequences are described in Table 1. These primers were synthesized by the Beijing Genomics Institute (BGI, Beijing, China).

Amplifications were performed in a 20 μL reaction containing the purified genomic DNA on one 1.2 mm punch of FTA card, 2 µL of 10 × PCR buffer, 0.25 μM of each primer, 150 μM of each dNTP (Bioline, London, UK), 2.5 mM of Mg^2+^, 0.5 U of *Taq* DNA polymerase (Qiagen, Hilden, Germany), and ddH_2_O to make up volume. The thermal profile for the amplification of the two regions consisted of an initial denaturation for 2 min at 94 °C, followed by 35 cycles of 30 s at 94 °C, 30 s at the annealing temperatures shown in Table 1, and 30 s at 72 °C, with a final extension of 5 min at 72 °C. Amplification was carried out in S1000 thermal cyclers (Bio-Rad, Hercules, CA, USA).

The amplicons produced were visualized by electrophoresis in 1% agarose gels using 1× TBE buffer (89 mM Tris, 89 mM Boric acid, and 2 mM Na_2_EDTA). Next, a 0.7 μL aliquot of each amplicon was mixed with 10 μL of loading dye (98% formamide, 10 mM EDTA, 0.025% bromophenol blue, and 0.025% xylene-cyanol). After denaturation at 95 °C for 5 min, the samples were cooled rapidly on wet ice and loaded onto 16 cm × 18 cm 14% acrylamide:bisacrylamide (37.5:1) (Bio-Rad) gels. Electrophoresis was performed using Protean II xi cells (Bio-Rad) for 18 h in 0.5 × TBE buffer. The gels were silver-stained by Byun et al. method [21].

### 2.3. DNA Sequencing and Sequence Analyses

PCR amplicons representative of different SSCP patterns from the Gannan yaks that appeared to be homozygous were directly sequenced in both directions using Sanger sequencing at the Beijing Genomics Institute (BGI). Banding pattern variants, which were only found in a heterozygous state, were isolated for sequencing (as previously described) using an approach described by Gong et al. [22].

Sequence alignments, translation, and comparisons were carried out using DNAMAN (version 5.2.10, Lyn-non BioSoft, Vaudreuil, QC, Canada). The BLAST algorithm was used to search the NCBI GenBank (http://www.ncbi.nlm.nih.gov/) databases.

### 2.4. Haplotype Determination

To ascertain the *FASN* haplotypes, progeny that typed as homozygous in either of the regions could have their haplotypes directly inferred based on the co-inheritance of sequences. For example, for an animal presenting with genotype *A_1_A_1_* for the region spanning exon 24-intron 24 and a genotype *A_2_B_2_* for the region spanning exon 34, the presence of haplotypes *A_1_-A_2_* and *A_1_-B_2_* could be directly inferred. If progeny typed as heterozygous in both regions, their haplotypes and diplotypes were not analyzed. This reduced the number of yaks studied in the haplotype and diplotype association analyses.

### 2.5. Statistical Analyses

All analyses were performed using IBM SPSS Statistics version 22.0 (IBM, New York, NY, USA). General Linear Mixed-Effects Models (GLMMs) were used to assess whether the presence or absence (coded as 1 or 0, respectively) of *FASN* haplotypes were associated with various milk traits in the Gannan yaks studied. Any haplotype that had associations in the single-haplotype models with a *p* value of less than 0.2 (*p* < 0.20), and thus could potentially impact the trait, were factored into the models. From that, we could determine the independent haplotype effects. In addition, for diplotypes with a frequency greater than 5% (thus providing an adequate sample size), a second set of GLMMs were used to ascertain the effect of diplotype on various milk traits. To reduce the probability of false positive results during the multiple comparisons undertaken in these models, a Bonferroni correction was applied.

Group and parity were found to affect milk traits, and they were accordingly fitted in the models. Unless otherwise indicated, all *p* values less than 0.05 were considered to be significantly different.

### 2.6. RNA Extraction and RT-qPCR Analysis

Using yak β-actin as an internal control, reverse transcription-qPCR (RT-qPCR) was performed to investigate yak *FASN* expression in different tissues. Two pairs of qPCR primers were designed to amplify a 203-bp fragment of yak *FASN* mRNA and a 133-bp fragment of the β-actin mRNA sequences (GenBank accession nos. XM_005902498 and DQ838049.1, respectively) (Table 1). Total RNA was extracted from the yak tissue samples using TRIzol reagent (Invitrogen, Carlsbad, CA, USA) according to the manufacturer’s instructions. The integrity and concentration of total RNA was assessed using 2% agarose gels in electrophoresis and UV spectrophotometry. The cDNA was synthesized by reverse transcription from the total RNA using the PrimeScript^TM^ RT Reagent Kit with the gDNA Eraser (Takara) according the manufacturer’s instructions. The amplification of the cDNA was conducted in 20 µL reaction mixture consisting of 100 ng cDNA, 0.25 µM of each primer, 10.0 µL AceQ qPCR SYBR^®^ Green Master Mix (Vazyme, Nanjing, China), 0.4 µL ROX Reference Dye 2, and made up with ddH_2_O to a volume of 20 µL. The thermal profiles were: One cycle of 5 min at 95 °C, followed by 40 cycles of 10 s at 95 °C, 30 s at 60 °C, and 30 s at 72 °C. Amplification was performed in Applied Biosystems Quant Studio^®^6 Flexq (Applied Biosystems, Carlsbad, CA, USA) and the 2^−∆∆*C*T^ method was used to analyze the relative level of gene expression [23].

## 3. Results

### 3.1. Identification of Sequence Variation in Yak FASN

**Figure 1:**
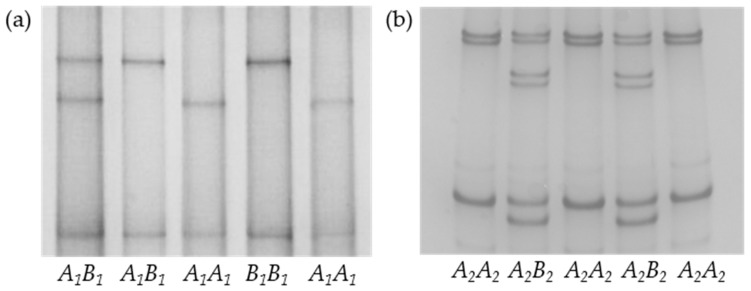
Polymerase Chain Reaction Single-Stranded Conformational Polymorphism (PCR-SSCP) banding patterns for the two regions of yak Fatty acid synthase gene (*FASN*) analyzed. (**a**,**b**) represent the *FASN* exon 24-intron 24 and exon 34 amplification regions, respectively.

**Figure 2:**
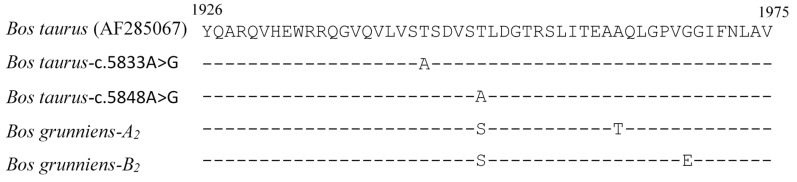
Alignment of the predicted amino acid sequences encoded by *FASN* exon 34 from *Bos taurus* and *Bos grunniens*. Amino acid sequences are presented in one letter code and slashes indicate sequences identical to the top sequence. Numbers above the sequences represent the amino acid positions.

Upon PCR-SSCP analysis of the 290 yaks, different SSCP patterns were obtained, and these suggested that there were two distinct variants for each of the two regions. These were named *A_1_* and *B_1_*, and *A_2_* and *B_2_*, for the exon 24-intron 24 and exon 34 regions, respectively. Upon sequencing, these were confirmed to represent two different DNA sequences for each region (Figure 1). The *A_1_* and *A_2_* sequence were identical to a reported yak *FASN* sequence (GenBank accession no. NW_005395032), whereas the sequences of *B_1_* and *B_2_* were both 99% homologous to the NW_005395032 sequence. This sequence variation was the result of the presence of three nucleotide substitutions: c.4296+38C/T (intron 24), c.5884A/G, and c.5903G/A, both located in exon 34. The exon 34 substitutions would result in the amino acid substitutions p. Thr1962Ala and p.Gly1968Glu if expressed (Figure 2).

Genotype, variant, and haplotype frequencies for the two regions of *FASN* in Gannan yaks are shown in Table 2. Genotypes *A_1_B_1_* (exon 24-intron 24 region) and *A_2_A_2_* (exon 34 region) were the most common genotypes in the two regions, with frequencies of 51.08% and 56.21%, respectively. A total of four haplotypes were identified, which spanned intron 23 to exon 34. Of these haplotypes, *A_1_-A_2_*, *B_1_-A_2_*, and *A_1_-B_2_* were the most common, with frequencies of 49.71%, 37.86%, and 8.96%, respectively. The *B_1_-B_2_* haplotype was rare (3.47%).

### 3.2. Association of Yak FASN Haplotype and Diplotype with Milk Traits

Associations were detected between the *FASN* haplotypes and milk traits. In the single-haplotype (presence/absence) models, the presence of haplotype *A_1_-A_2_* was associated with increased milk fat percentage (*p* = 0.050) and increased total milk solid percentage (*p* = 0.037) (Table 3). These associations remained significant when the other haplotypes were factored into the models. In these latter models, haplotypes *A_1_-B_2_* and *B_1_-B_2_* was also found to be associated with reduced milk fat percentage, while *B_1_-B_2_* was found to be associated with reduced total milk solid percentage. No associations with milk protein percentage, non-fat milk solid percentage, or milk lactose percentage were detected for any haplotype.

The diplotypes *A_1_-A_2_*/*A_1_-A_2_* (*n* = 41), *A_1_-A_2_*/*A_1_-B_2_* (*n* = 31), *A_1_-A_2_*/*B_1_-A_2_* (*n* = 59), *B_1_-A_2_*/*B_1_-A_2_* (*n* = 30), and *B_1_-A_2_*/*B_1_-B_2_* (*n* = 12) occurred at a frequency of greater than 5.0% in the yaks that could be diplotyped. The *FASN* diplotype was found to have an effect on milk fat percentage (*P* = 0.038), with yaks with the *A_1_-A_2_*/*B_1_-A_2_* diplotype having a higher marginal mean milk fat percentage (5.4 ± 2.43%), than those with the diplotype *B_1_-A_2_*/*B_1_-B_2_* (4.1 ± 1.96%; Table 4). Diplotype was not found to have a significant effect on milk protein percentage, non-fat milk solid percentage, or milk lactose percentage, but a trend was evident (*p* = 0.051) with the *A_1_-A_2_*/*B_1_-A_2_* diplotype, which had a higher marginal mean total milk solid content than the other diplotypes (Table 4).

### 3.3. Expression of Yak FASN in Different Tissues

**Figure 3:**
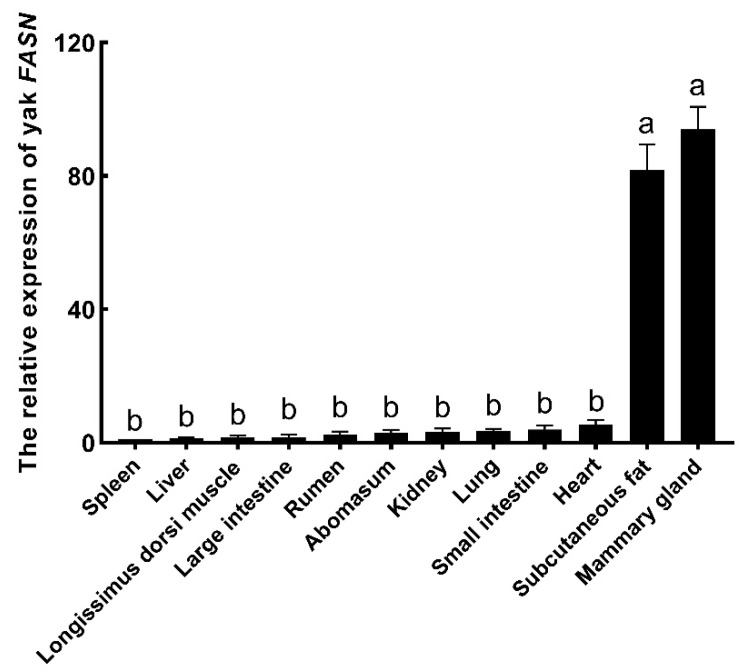
The relative expression of *FASN* in twelve yak tissues. The spleen was chosen as the control tissue. The error bars indicate standard errors. The different lower-case letters above the columns indicate significant differences (*p* < 0.01).

The *FASN* expression levels were different in the yak tissues (*p* < 0.01; Figure 3). The highest expression was found in the mammary gland, followed by subcutaneous fat, and only low levels of expression were observed in the other tissues, including the heart, small intestine, lung, kidney, abomasum, rumen, large intestine, *longissimus dorsi* muscle, and liver.

## 4. Discussion

Yak milk is widely used to produce butter and ghee products, and these constitute a major component of Qinghai–Tibetan pastoralists’ and nomadic peoples’ daily food intake. Compared with *B. taurus* milk, yak milk contains fat in the range of 5.3%–8.8% (*w*/*v*), which is almost twice that of taurus cow milk [4], but the yield of yak milk is lower at approximately 10% of the yield of a taurus dairy cow milk [1].

This is the first report describing the association between variation in *FASN* and milk fat traits in Gannan yaks, but in an earlier study, g.5477C/T in intron 3 of Datong yak *FASN* was associated with an increase in intramuscular fat content [19]. The two coding region substitutions c.5884A/G and c.5903G/A report here are located in yak *FASN* exon 34, and would, if expressed, result in the amino acid substitutions p.Thr1962Ala and p.Gly1968Glu, respectively. Roy et al. [11] described a nucleotide substitution c.5833A/G (originally g.16009A/G) in exon 34 of the cow *FASN* gene, which would result in a threonine to alanine substitution (p.Thr1945Ala), and showed that it was associated with increased milk fat content. This change is in a domain of the mature protein that has enoyl reductase (ER) and ketoacyl reductase (KR) activity. Abe et al. [9] described two other nucleotide substitutions in this exon of *FASN*, c.5848A/G (originally g.16024A/G) that would, if expressed, produce a threonine to alanine substitution at position 1950 of the mature protein (p.Thr1950Ala), and c.5863T/C (originally g.16039T/C), that would produce p.Trp1955Arg. Their research was conducted on Japanese Black cattle, and while they revealed associations between these exon 34 nucleotide substitutions and the FA composition of back-fat, intermuscular fat, and intramuscular fat, they did not analyze milk.

Later in 2009, Schennink et al. [14] also reported variation in *FASN* and its effect on bovine milk composition. While they appear to have erroneously assumed that g.16009A/G (c.5833A/G) and g.16024A/G (c.5848A/G) were the same nucleotide substitutions, they nevertheless concluded that the exon 34 region of *FASN* was associated with variation in C14:0 FA levels in the *B. taurus* cows’ milk. Matsumoto et al. [15] investigated c.5848A/G (p.Thr1950Ala) in Holstein cattle and reported that the sequence variation had an effect on milk fat content and the C14 index. More recently, Mauric et al. [24] associated the substitutions described by Abe et al. [9] with milk fat content and total milk fat in Holstein × Simmental cross cattle, but reported a breed by genotype interaction. Taken together, this would suggest that exon 34 of *FASN* affects milk fat traits, which is likely to be as a consequence of some effect on FASN enoyl reductase (ER) and ketoacyl reductase (KR) activity in a way that is probably similar in both *B. taurus* and *B. grunniens*. An alignment of the amino acid sequences in this exon 34 region (Figure 2) would support that contention.

Morris et al. [12] also revealed that two sequence variations, c.5572-18G/A in intron 32 (originally reported as g.15603G>A in intron 31) and c.6414-72_6414-71delinsA in intron 37 (originally reported as g.17250_17251ATindel in intron 36), were associated with milk fat content and the percentage of C14:0 in subcutaneous adipose tissue in dairy cattle. While c.4296+38C>T in this study is also not located in the coding region, it may nevertheless affect *FASN* expression. Intron variation can, for example, affect transcription efficiency by altering the sequence of regulatory elements such as enhancers, silencers, and other DNA structures [25]. In this respect, DNA sequence variation in intron 1 of rat *FASN* has been reported to be associated with tissue specific *FASN* expression [26]. This suggests that the intron contains a tissue-specific response element of some kind.

In Murrah buffaloes (*Bubalus bubalis*), Kumar et al. [27] identified that variation in exon 40 of *FASN*, a region containing a thioesterase domain, is associated with milk fat traits, including lactation fat average and lactation total solid average. This suggests that this domain of FASN has a similar effect on milk fat traits as the variation reported here in the haplotypes spanning the exon 24-intron 24 to exon 34 region in yaks. The comparison is notable because buffalo milk fat percentage levels (7.9%–8.4%) are closer to those observed in yaks than they are in *B. taurus* cattle.

The *FASN* gene is expressed in a variety of tissues in mammals. In this study, we found that the highest expression was in the yak mammary gland, followed by subcutaneous fat tissue. The expression was weak in the heart, small intestine, lung, and kidney. Yaks in the Qinghai–Tibet Plateau live at an altitude of 2000 to 5000 m, and the climate in this region can be very cold over winter. In order to cope with this cold, they deposit subcutaneous fat prior to the winter [1], and thus adipogenesis would be expected to increase at that time of year. This would be consistent with the results obtained here, where, aside from the mammary gland, higher levels of expression of *FASN* were only seen in the subcutaneous fat. High levels of *FASN* gene expression have previously been reported in lactating and non-lactating buffalo (*B. bubalis*) mammary gland and subcutaneous fat tissue [28], although in humans, *FASN* was reported to be expressed in all tissues studied, with the highest levels observed in the liver and lungs [29].

## 5. Conclusions

This study enriched our knowledge of the tissue expression of the yak FASN gene and also revealed variation in the gene. Yak *FASN* was highly expressed in the mammary gland and subcutaneous fat. Variants and haplotypes of exon 24-intron 24 and exon 34 of yak *FASN* were associated with the milk fat percentage and total milk solid percentage. These results suggest that *FASN* variation could be used as a genetic marker in breeding programs to improve milk fat content and total milk solid levels in yaks.

## Figures and Tables

**Table 1:** Polymerase chain reaction (PCR) primers used in this study.

Gene	Region	Primer Sequence (5′-3′)	Amplicon Size (bp)	Purpose of Primers
*FASN*	Exon 24-intron 24	F: CTGTCACCTTCCTCACTTGCCCT	390	PCR-SSCP analysis
		R: GAGGAGGAATCGGCCAGGATGTT		
*FASN*	Exon 34	F: CCCTCTAAAGCCGTCCTCACCA	220	PCR-SSCP analysis
		R: CCAGACCTTCATTTGCCAATCCTC		
*FASN*		F: ACAAGACAAGCCCGAGGAG	203	RT-qPCR
		R: TAGCAGGCAGTTCCGAGAG		
β-actin		F: AGCCTTCCTTCCTGGGCATGGA	113	RT-qPCR
		R: GGACAGCACCGTGTTGGCGTAGA		

bp: base pairs; PCR-SSCP: polymerase chain reaction-single stranded conformational polymorphism; RT-qPCR: reverse transcription-quantitative PCR.

**Table 2:** Genotype, variant, and haplotype frequency of yak *FASN.*

Genotype Frequency (%)					Variant Frequencies (%)				Haplotype Frequencies (%)			
Exon 24-Intron 24			Exon 34		Exon 24-Intron 24		Exon 34		Exon 24-Intron 24 to Exon 34			
*A_1_A_1_*	*A_1_B_1_*	*B_1_B_1_*	*A_2_A_2_*	*A_2_B_2_*	*A_1_*	*B_1_*	*A_2_*	*B_2_*	*A_1_*-*A_2_*	*A_1_*-*B_2_*	*B_1_*-*A_2_*	*B_1_*-*B_2_*
31.60	51.08	17.32	56.21	43.79	57.00	43.00	78.00	22.00	49.71	8.96	37.86	3.47

**Table 3:** Association between the presence/absence of *FASN* haplotypes and milk traits (Mean ± SE) ^1^ in Gannan yaks.

Traits	Haplotype Assessed	Other Haplotypes in Model	Mean ± SE				*p* Value
			Present	*n*	Absent	*n*	
Milk protein (%)	*A_1_-A_2_*		4.9 ± 0.07	131	4.8 ± 0.12	42	0.421
	*A_1_-B_2_*		4.9 ± 0.14	31	4.9 ± 0.07	142	0.880
	*B_1_-A_2_*		4.9 ± 0.08	101	4.9 ± 0.09	72	0.978
	*B_1_-B_2_*		4.7 ± 0.38	10	4.9 ± 0.81	163	0.363
Milk fat (%)	*A_1_-A_2_*		4.9 ± 0.18	131	4.2 ± 0.31	42	**0.050**
	*A_1_-B_2_*		4.3 ± 0.36	31	4.9 ± 0.17	142	0.154
	*B_1_-A_2_*		4.9 ± 0.20	101	4.6 ± 0.24	72	0.300
	*B_1_-B_2_*		3.7 ± 1.53	10	4.8 ± 2.04	163	0.081
	*A_1_-A_2_*	*A_1_-B_2_*, *B_1_-B_2_*	4.9 ± 2.11	131	4.2 ± 1.69	42	**<0.001**
	*A_1_-B_2_*	*A_1_-A_2_*, *B_1_-B_2_*	4.3 ± 1.70	31	4.8 ± 2.09	142	**<0.001**
	*B_1_-B_2_*	*A_1_-A_2_*, *A_1_-B_2_*	3.7 ± 1.53	10	4.8 ± 2.04	163	**<0.001**
Milk total solid (%)	*A_1_-A_2_*		16.0 ± 0.21	131	15.1 ± 0.37	42	**0.037**
	*A_1_-B_2_*		15.4 ± 0.43	31	15.9 ± 0.20	142	0.378
	*B_1_-A_2_*		15.9 ± 0.24	101	15.6 ± 0.29	72	0.351
	*B_1_-B_2_*		14.5 ± 1.27	10	15.9 ± 2.45	163	0.086
	*A_1_-A_2_*	*B_1_-B_2_*	16.0 ± 2.49	131	15.1 ± 2.60	42	**<0.001**
	*B_1_-B_2_*	*A_1_-A_2_*	14.5 ± 1.27	10	15.9 ± 2.45	163	**<0.001**
Non-fat solid (%)	*A_1_-A_2_*		11.1 ± 0.11	131	10.9 ± 0.19	42	0.282
	*A_1_-B_2_*		11.2 ± 0.22	31	11.0 ± 0.10	142	0.567
	*B_1_-A_2_*		11.0 ± 0.12	101	11.1 ± 0.14	72	0.844
	*B_1_-B_2_*		10.9 ± 0.80	10	11.1 ± 1.23	163	0.590
Milk lactose (%)	*A_1_-A_2_*		4.9 ± 0.61	131	4.9 ± 0.49	42	0.313
	*A_1_-B_2_*		5.0 ± 0.10	31	4.9 ± 0.05	142	0.319
	*B_1_-A_2_*		4.9 ± 0.06	101	4.9 ± 0.07	72	0.647
	*B_1_-B_2_*		4.9 ± 0.62	10	4.9 ± 0.57	163	0.806

^1^ Estimated marginal means and standard errors (SE) of those means derived from general linear mixed-effects models with parity and group included in the models as fixed and random factors, respectively. *p* < 0.05 is in bold.

**Table 4:** Association of *FASN* diplotypes with milk traits (Mean ± SE)^1^ in Gannan yak.

Milk Traits	Mean ± SE					
	*A_1_-A_2_/A_1_-A_2_*	*A_1_-A_2_/A_1_-B_2_*	*A_1_-A_2_/B_1_-A_2_*	*B_1_-A_2_/B_1_-A_2_*	*B_1_-A_2_/B_1_-B_2_*	*p* Value
	*n* = 41	*n* = 31	*n* = 59	*n* = 30	*n* = 12	
Milk protein (%)	4.9 ± 0.97	4.9 ± 0.69	5.0 ± 0.80	4.8 ± 0.71	4.9 ± 0.79	0.901
Milk fat (%)	4.8 ± 1.75 ^ab^	4.3 ± 1.70 ^ab^	5.4 ± 2.43 ^a^	4.3 ± 1.60 ^ab^	4.1 ± 1.96 ^b^	**0.038**
Milk total solid (%)	15.7 ± 2.13	15.4 ± 2.13	16.5 ± 2.81	15.1 ± 2.08	15.1 ± 2.10	0.051
Non-fat solid (%)	11.0 ± 1.72	11.2 ± 0.90	11.2 ± 1.05	10.8 ± 1.08	11.0 ± 0.82	0.775
Milk lactose (%)	4.9 ± 0.83	5.0 ± 0.44	4.9 ± 0.46	4.8 ± 0.47	4.9 ± 0.59	0.752

^1^ Estimated marginal means and standard errors (SE) of the means derived from general linear mixed-effects models with parity and group included in the models as fixed and random factors, respectively. Means within rows that do not share a superscript letter (a or b) are significantly (*p* < 0.05) different and bolded.
